# Integrative Advances in Pig Genomics: From Reference Assemblies and Evolutionary History to the Mechanistic Dissection of Key Traits

**DOI:** 10.3390/biology15050447

**Published:** 2026-03-09

**Authors:** Shengguo Tang, Dongfang Li, Ying Lu, Zhendong Gao, Bo Wang, Xingneng Liu, Hongjiang Wei, Jiao Wu

**Affiliations:** 1 Yunnan Provincial Key Laboratory of Animal Nutrition and Feed, Faculty of Animal Science and Technology, Yunnan Agricultural University, Kunming 650201, China; 15802587908@163.com (S.T.); dfli0927@163.com (D.L.); yinglu_1998@163.com (Y.L.); zander_gao@163.com (Z.G.); wangbo@ynau.edu.cn (B.W.); 18787055931@163.com (X.L.); 2 Yunnan Provincial Key Laboratory of Development and Utilization of Plateau Characteristic Circular Agriculture Resources, Institute of Yunnan Circular Agricultural Industry, Pu’er 665000, China; 3 Yunnan Provincial Key Laboratory for Porcine Gene Editing and Xenotransplantation, Yunnan Agricultural University, Kunming 650500, China

**Keywords:** pig, genomics, pangenome, structural variation, economic traits, breeding applications

## Abstract

Pigs are a major source of animal protein and an important model for studying domestication, adaptation, and the genetics of complex traits. Over the past decade, pig genomics has progressed from generating reference assemblies and variant catalogues toward reconstructing population history and interpreting phenotypic divergence with greater resolution. Improvements in reference continuity, breed-matched assemblies, and pangenome/graph representations reduce reference bias in repeat-rich and structural-variant-prone regions, strengthening cross-population comparisons and fine-mapping across cohorts. Time-stratified ancient genomes provide an explicit temporal framework for evaluating lineage turnover and gene flow and support a multi-stage, network-based view of domestication. In parallel, genetic analyses of growth, carcass composition, meat quality, and disease resilience increasingly integrate association signals with regulatory annotation, gene expression, and tissue- or stage-specific context. Across these lines of work, maintaining comparability across reference frameworks remains central, including stable coordinate systems, robust imputation resources, and reproducible approaches for structural-variant genotyping. Together, these developments support more consistent inference and interpretation while providing a clearer basis for translation in breeding and biomedical research with attention to genetic diversity.

## Simple Summary

Pig genomics now serves two main uses: improving pig breeding and supporting biomedical research. Many key variants lie in repeat-rich regions or involve structural changes, where older references and linear pipelines can lose sequence, place reads incorrectly, or miss real haplotypes. New assemblies that are breed-specific, near gap-free, and increasingly telomere to telomere reduce these problems and improve routine variant detection. Pangenomes and graph approaches also help by capturing sequences absent from a single reference, which makes comparisons across breeds and cohorts more reliable. Ancient DNA and time-resolved data further show that domestication involved repeated movements and gene flow, which affects how we interpret selection signals. For economic traits, the focus is shifting from locating signals to explaining what they change by linking associations to regulatory annotation and tissue or stage-specific expression. Consistent coordinates and variant definitions are essential for results that can be reproduced and applied in breeding.

## 1. Introduction

Pork is a central component of the global meat supply and food security [1]. In 2022, it represented about one-third of global meat consumption. Accordingly, even modest shifts in pig supply or demand can ripple into feed markets and trade flows, amplify price volatility, and expose supply chain fragility [2]. The Organization for Economic Co-operation and Development/Food and Agriculture Organization of the United Nations(OECD/FAO) projections place global pork consumption at around 131 million tons (carcass weight equivalent) by 2033 [3]. In debates on sustainable and healthy diets, pork is frequently regarded as an affordable source of high-quality protein and essential micronutrients. Consequently, it occupies a central position at the intersection of nutrition, affordability, and environmental trade-offs [4].

Shock sensitivity differs across countries because production and consumption are large in absolute terms but heterogeneous in structure. Pork and poultry jointly provide the dominant animal source protein in many diets, but substitution elasticities and consumption profiles vary widely across regions [2,5]. Production is also concentrated: reports for 2024 to 2025 attribute roughly 49 percent of global output to China, about 18 percent to the European Union, and about 11 percent to the United States [6]. That concentration means animal health events, trade disruptions, or feed cost spikes in a few major producers can propagate quickly and show up as cross-regional volatility [7].

Pigs also occupy a distinctive position in biomedicine as large animal models. Compared with small laboratory species, their body size, organ anatomy, physiology, and immune features more closely resemble humans, enabling translational studies spanning cardiovascular and metabolic disease, transplantation and device testing, infection and immunity, and the gut microbiome [8,9,10]. Progress in pig genomics, therefore, informs both breeding and the genomic resources and functional annotation needed for disease control and biomedical innovation [11].

From an evolutionary perspective, domestication and dispersal in *Sus* reflect a network history rather than a linear narrative. Eurasian wild boar lineages were shaped by repeated movements, isolation, and secondary contact, with genome-wide evidence consistent with demographic contractions and expansions across glacial cycles and the persistence of multiple lineages [12]. Domestication likely involved more than one geographic unit, and subsequent patterns are best explained by sustained gene flow and recurrent backcrossing, yielding protracted admixture across regions [13]. Ancient DNA provides the necessary temporal resolution, including near genome-wide ancestry turnover in pigs introduced into Europe, with regional variation in the pace and magnitude of turnover linked to husbandry practices and wild boar recruitment [14]. With improved genome resources and haplotype-resolved methods, signals of domestication and adaptation can be localized more precisely, making it easier to connect historical processes with trait divergence [15]. Genomic analyses of human-mediated transport systems, including islands, further support repeated introductions, followed by admixture, shaping present day structure [16].

Industrial breeding has intensified selection and can narrow the genetic base over short timescales, elevating inbreeding and diversity loss risks [17,18]. While genomic selection improves response, durable programs typically require explicit constraints on inbreeding and diversity [18]. Three-way cross systems increase uniformity but may accelerate homogenization; changes in terminal sire usage can also shift growth, carcass composition, and meat quality in expected directions [19]. In China, the Duroc–Landrace–Yorkshire system dominates and is estimated to contribute at least 70 percent of pork output [20,21]. Although local breeds retain distinctive haplotypes and substantial variation, their structure is rapidly reshaped by industrialization and introgression, motivating optimization frameworks such as optimal contribution selection to balance gain, inbreeding rate, and external contributions [22,23]. Under African swine fever and other persistent disease pressures, resilience has become a central breeding objective, supported by emerging evidence that host genetic variation can influence infection outcomes [24,25].

In pigs, conclusions about domestication and complex traits increasingly rely on the representation and comparison of genetic variation across different reference frames. We summarize how the field moved beyond a single linear reference to breed-matched assemblies and pangenome/graph resources, making more sequence and structural variation accessible. Interpreting associations requires linking variant representations to regulatory annotation and expression. The goal is to specify the affected element, the likely target gene, and the tissue or developmental stage where the effect is measurable. This is most acute in repeat- and SV-rich regions, where reference choice changes read placement and breakpoint resolution, altering both SV discovery and fine-mapping results. Our goal is an inference that is both reproducible and portable across datasets and breeding settings, with a clear route to testing and application.

## 2. Building and Iterating Pig Reference Genomes

This section follows the technical logic behind pig references, from early drafts to chromosome-scale assemblies and, more recently, toward near gap-free and telomere-to-telomere (T2T) sequences. The aim is practical. Gaps and structural errors do not stay confined to the assembly. They change read placement, distort variant calling, and shift functional annotation, and those effects can cascade into downstream interpretation. We also discuss breed-specific assemblies and the rise of pangenome and graph frameworks, focusing on what they change for GWAS, structural variant (SV) discovery, and breeding inference. A recurring theme is reproducibility. If reference choice and processing steps are not fully specified, differences between studies can reflect coordinate and representation effects as much as biology.

### 2.1. Evolution of Pig Reference Assemblies

In 2012, the Swine Genome Sequencing Consortium released the first widely adopted pig reference, built from a purebred Duroc female using a BAC clone map combined with whole-genome shotgun sequencing. Sscrofa10.2 anchored about 2.60 Gb to chromosomes and left roughly 212 Mb in unplaced scaffolds, with scaffold and contig N50 values of around 0.64 Mb and 80.7 kb. It created a shared coordinate system, but repeat-rich and structurally complex regions still contained gaps and misassemblies, which are often where linear references first become unreliable in real analyses [26].

Later updates were driven by two recurring issues: a missing sequence and an incorrect structure. One objective was to reduce fragmentation and close gaps so that reads align more uniformly and gene models are less affected by assembly breaks. The other was to correct misjoins and collapsed repeats in repeat dense regions, including telomeric and centromeric neighborhoods, so that coordinates remain interpretable within complex sequence contexts and across diverse genetic backgrounds [27]. These changes matter because errors in repeat-dense regions tend to propagate. They bias alignments, perturb single-nucleotide polymorphism (SNP) and SV calling, and can shift gene models or regulatory annotations, creating cross-study differences that look biological on paper. Reference bias is, therefore, a reproducibility problem, not a minor technical nuisance [28,29].

As PacBio and Oxford Nanopore Technologies sequencing (ONT) long reads, together with chromosome-scale scaffolding such as High-throughput Chromosome Conformation Capture (Hi-C), became routine in livestock genomics, pig references improved in contiguity and base-level accuracy while better-preserving the repeat structure and gene space [30,31,32]. These improvements have several concrete downstream effects: fewer loci are located within assembly gaps or misassembled regions, fewer sequencing reads map ambiguously to repetitive regions, and coordinate consistency is enhanced when comparing different cohorts. That makes population structure analyses, GWAS peaks, and selection signals easier to reconcile across studies when datasets are aligned to the same reference and annotation backbone [33,34]. Updated references, such as Sscrofa11.1, also reduced fragmentation and improved annotation consistency, which supports integrative analyses that combine WGS with RNA-seq and ATAC-seq and reduce sensitivity to reference choice when results are contrasted across projects [35].

**Figure 1:**
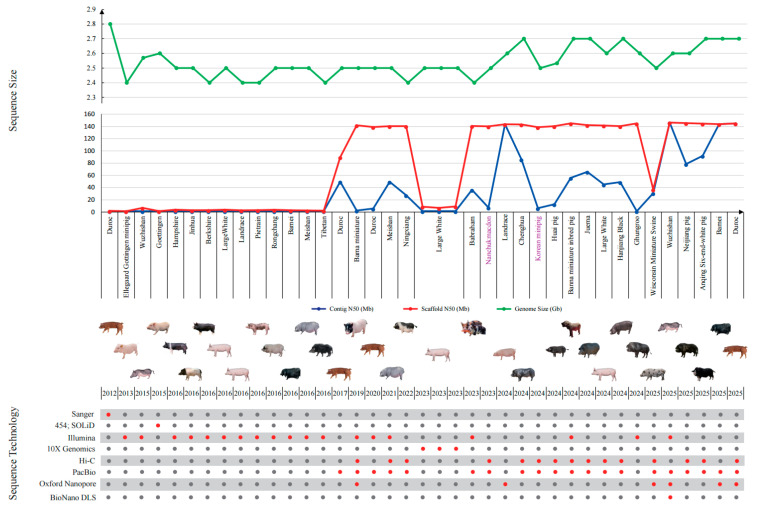
Timeline of reference assembly iteration. Specifically, red dots denote sequencing technologies applied in the corresponding assembly, whereas grey dots indicate technologies that were not used.

Two reported near-gap-free or T2T-level assemblies further illustrate recent progress toward improved genome continuity and structural resolution in pigs. Here, near-T2T refers to assemblies approaching telomere-to-telomere continuity that exhibit high contig and scaffold continuity together with substantial gap reduction in repeat-rich regions, enabling more stable read mapping and improved structural variant representation [36]. To make the link between assembly technology and reference evolution easier to follow, we provide a summary of representative pig references and near-T2T assemblies from the past decade in Table 1 and Supplementary Table S1. These tables include 39 references, near-T2T, and T2T assemblies published between 2012 and 2025, spanning about 29 breeds. They enable a side-by-side comparison of technical routes and continuity improvements, highlighting the progress toward more complete genome representation. Assembly continuity metrics, such as N50 and BUSCO, provide complementary indicators of genome quality. Higher N50 values reflect improved assembly continuity and facilitate read mapping and structural variant detection, while BUSCO scores indicate conserved gene completeness and annotation reliability. However, these metrics do not directly measure the resolution of repeat-rich or structurally complex regions and should, therefore, be interpreted as informative but partial indicators when evaluating assemblies for repeat- and SV-related analyses. We also provide a paired timeline schematic in Figure 1 that connects methodological transitions to the problems they addressed, so readers can track why each version mattered for analysis rather than memorizing version labels. Recent examples, including gap-free assemblies from Chinese local lineages and near-T2T assemblies for breeds such as the Jinhua pig, illustrate how increased continuity can stabilize read mapping, improve variant representation, and enhance interpretability in repeat-rich genomic landscapes [37,38]. In practice, the best evidence for these gains is not a single continuity statistic, but more consistent DNA and RNA mapping and cleaner variant representations across lineages [35].

Preprints pursuing a single gap-free T2T pig reference are now emerging, and they sit alongside a second trajectory that is expanding multi-reference and graph representations to capture population diversity more directly [37,39]. In this review, we interpret each reference update by what it makes newly analyzable, especially SV discovery, gene gain or loss, and functional elements embedded in complex sequences.

**Table 1:** Iteration of pig reference genomes and representative near-T2T assemblies.

Breed	Genome Versions	Assembling Evaluation Metrics			Year	Reference
		Contig N50/Mb	Scaffold N50/Mb	BUSCO/%		
Duroc	Sscrofa10.2	0	0.576	--	2012	[26]
Ellegaard Gottingen minipig	SscrofaMinipig	0.022	0.022	--	2013	GCA_000331475.1
Wuzhishan	minipig_v1.0	0.0235	5.432	--	2015	[40]
Goettingen	ss10.2_mar2013	0.0173	0.154	--	2015	GCA_001292865.1
Hampshire	Hampshire_pig_v1	0.0888	2.4	--	2016	GCA_001700165.1
Jinhua	Jinhua_pig_v1	0.0952	1.5	--	2016	GCA_001700295.1
Berkshire	Berkshire_pig_v1	0.0947	1.7	--	2016	GCA_001700575.1
LargeWhite	Large_White_v1	0.0888	2.4	--	2016	GCA_001700135.1
Landrace	Landrace_pig_v1	0.0881	1.4	--	2016	GCA_001700215.1
Pietrain	Pietrain_pig_v1	0.0806	1.7	--	2016	GCA_001700255.1
Rongchang	Rongchang_pig_v1	0.0791	2.3	--	2016	GCA_001700155.1
Bamei	Bamei_pig_v1	0.0709	1.5	--	2016	GCA_001700235.1
Meishan	Meishan_pig_v1	0.0633	1.2	--	2016	GCA_001700195.1
Tibetan	Tibetan_Pig_v2	0.0572	0.8619	--	2016	GCA_000472085.2
Duroc	Sscrofa11.1	48.23	88.23	93.8	2017	[35]
Bama miniature	ASM764409v1	1	140.4	93.9	2019	[41]
Duroc	Ninghe_*Sus*_1	4.3	137.6	--	2020	GCA_015776825.1
Meishan	ASM1795798v1	48.05	138.97	--	2021	[42]
Ningxiang	ASM2056790v1	26.1	139	--	2022	[33]
Large White	ASM2978402v1	0.0933	7.3	--	2023	GCA_029784025.1
Large White	ASM2989022v1	0.0923	5.4	--	2023	GCA_029890225.1
Large White	ASM2989021v1	0.0822	7.8	--	2023	GCA_029890215.1
Babraham	TPI_Babraham_pig_v1	34.95	139.2	--	2023	[43]
Nanchukmacdon	NCMD	5.9	138.6	93.1	2023	[44]
Landrace	norwegian_landrace	142.1	142.1	--	2024	GCA_963921485.1
Chenghua	ASM3744751v1	84.7	141.8	--	2024	[45]
Korean minipig	ASM3965481v1	4.9	137.3	93.8	2024	[46]
Huai pig	CAU_Huaizhu_1.1	11.37	138.92	95.33	2024	[47]
Banna miniature inbred pig	Banna	54.5	143.6	96.3	2024	[34]
Juema	ASM4086911v1	64.4	140.7	98	2024	[48]
Large White	ASM4490610v1	43.8	140.1	97.9	2024	
Hanjiang Black	ASM4490618v1	47.5	138.9	97.8	2024	
Ghungroo	ICAR-NRCP-G1	0.0111	143.4	--	2024	GCA_046128825.1
Wisconsin Miniature Swine	UWBC_WMS_v1	29.5	35.5	98.2	2025	[49]
Wuzhishan	T2T_pig1.0	144.9	144.9	97.9	2025	[50]
Neijiang pig	NJ_2022_AUG	77.1	144	96.24	2025	[51]
Anqing Six-end-white pig	ASM5023112v1	90.5	143.1	98.67	2025	[52]
Bamei	ASM3070493v2	142.3	142.3	--	2025	GCA_030704935.2
Duroc	T2T-Sscrofa	143.5	143.5	--	2025	[39]

Note: Major pig reference assemblies published between 2012 and 2025 are summarized, including genome size and assembly continuity metrics relevant to evaluating progress toward near-T2T genome representation and cross-study comparability. BUSCO values are unavailable for some assemblies because they were not reported in the original publications or were generated using different BUSCO database versions, limiting direct comparison across datasets. Abbreviation: BUSCO, Benchmarking Universal Single-Copy Orthologs.

### 2.2. Breed-Specific Assemblies and Emerging Pig Pangenome Resources

A single linear reference remains useful because it anchors annotation and keeps cross-study comparisons tractable. Its weak points, however, coincide with the hardest parts of the pig genome: SV-rich loci, repeat-complex regions, and sequence missing from the reference individual [53]. Breed-specific assemblies tackle these gaps by rebuilding sequence and structure in a matched background. The payoff is practical: the regions that a generic reference compresses, misplaces, or omits become visible, and the locus architecture becomes easier to interpret when divergence is structural or lineage-specific [47,54].

Chromosome-scale assemblies for Chinese local breeds and biomedical model pigs have grown quickly in the past few years. High-fidelity sequencing (HiFi) or ONT reads paired with Hi-C (or related scaffolding) have turned repeat resolution and SV-ready contiguity into a routine pipeline outcome rather than a special-case achievement [55]. Assemblies for breeds such as the Huai pig and the Banna miniature inbred pig typically include synteny against Sscrofa11.1 and highlight concrete structural differences, so continuity gains translate into tractable, trait-facing hypotheses rather than only improved summary statistics [34,47].

Once the question shifts from one individual to population-scale variation, the ceiling of a single reference becomes harder to ignore. Non-reference sequence, presence–absence variation, and complex SVs are systematically undercounted in linear workflows because they map poorly and are hard to represent consistently in one coordinate system [56,57]. Human pangenomes illustrate the consequence. Relative to GRCh38, they add a substantial polymorphic sequence and surface SVs that are missed or poorly resolved under linear-reference analysis [58,59]. Pigs will differ in magnitude, but the direction is the same: one reference tends to underestimate the variable sequence space.

As breed-specific assemblies expand from isolated case studies into multi-breed collections, pangenome analysis becomes feasible. Instead of treating diversity as deviations from a single baseline, pangenomes quantify core, variable, and private genes directly, along with presence–absence variation, SVs, and non-reference sequence [53]. This is particularly helpful for comparing gene-family turnover and SV landscapes across lineages, while still offering an accessible entry point for trait mapping [60].

Representation is also shifting from parallel assemblies toward graph genomes. Graph coordinates can place SNPs and SVs within a unified representation, potentially reducing mapping and localization bias and improving the stability of variant descriptions for population inference and functional interpretation [61]. Graph-based genotyping further matters because it brings SV genotyping closer to the accuracy and scale required for reuse with large short-read cohorts and for routine pipelines, rather than remaining a boutique analysis step [62]. As graphs scale beyond dozens of assemblies, engineering constraints become a first-order issue. Compressed structures, such as mutation-annotated networks, offer one route to store and share large multi-breed resources without losing sequence context [63].

Pig pangenome studies are increasingly measuring reference bias directly. A pan-pig pangenome built from 250 individuals recovered 308.3 Mb of non-reference sequence beyond Sscrofa11.1 and reported 3438 genes absent from the reference [64]. Graph analyses using multiple assemblies reported about 105 Mb of non-reference sequence and showed that incorporating this sequence into a graph can improve read mapping and downstream variant interpretation in resequencing data [65]. Together, these results imply that SNP-centric workflows on one linear reference can miss a meaningful fraction of variable sequence, including content plausibly relevant to immunity or environmental adaptation [61].

Evidence from other livestock points in the same direction. In a global cattle graph pangenome, 40.14% of SVs were poorly tagged by nearby SNPs (linkage disequilibrium r2 < 0.6), suggesting many SVs behave as largely independent signals even in SNP-dense regions [66]. Simply increasing SNP density, therefore, will not reliably recover SV-driven associations. Application-oriented integration is also accelerating: graph resources are increasingly combined with population resequencing and functional genomics to connect SVs and presence–absence variation with expression regulation, immune gene-family dynamics, and adaptation signals within a unified interpretive framework [59].

Breed-specific assemblies raise sequence and structural accuracy in a matched background, while pangenomes and graphs aim to represent variable sequence space more completely at the population scale. They are best treated as complementary extensions of linear workflows, not replacements that apply everywhere [66]. In practice, cross-lineage comparisons, structurally complex gene families, and SV-driven hypotheses benefit most from breed-specific or graph-based references. SNP-heavy population summaries can still use a linear baseline, with graph steps introduced where structural representation becomes limiting [65]. The advantages extend beyond simply increasing the number of detected variants. A more stable reference representation reduces coordinate inconsistencies across cohorts and analytical pipelines, thereby improving reproducibility in SV genotyping, ancestry decomposition, and fine mapping. From an analytical standpoint, genome representation should match study objectives. Sscrofa11.1 is sufficient for routine SNP-based analyses, genomic prediction, and compatibility with legacy datasets. Breed-specific assemblies are preferable when structural variation or a lineage-specific sequence is central, whereas pangenome or graph representations are most informative for multi-breed and SV-aware analyses requiring simultaneous haplotype representation. In practice, linear references suit SNP-dominated analyses, while breed-matched or graph-based frameworks become necessary when structural diversity, a non-reference sequence, or cross-lineage comparability are primary analytical targets.

### 2.3. Technical Implications for Downstream Analyses

Reference updates change analyses in a predictable order: read placement first, then variant detectability, and finally the pattern of systematic bias introduced by gaps and misassemblies [38]. Recovering a repeat-rich sequence, especially in telomeric and centromeric neighborhoods, can improve coverage uniformity, sharpen breakpoint localization, and reduce spurious SV signals that are, in fact, reference artifacts rather than true polymorphism [39]. This matters in pigs because gene families linked to immunity, adaptation, and production are often embedded in structurally complex regions. If the reference is incomplete or misassembled there, detection drops immediately, and annotation-based mechanism inference can be pulled off target because the underlying sequence context is distorted [43].

Equally important, mapping the same dataset to different references rarely yields fully comparable results. Coordinates can shift, allele encodings can change, and SV call sets can diverge, especially when updates close gaps, rebuild repeats, or alter how structural alleles are represented. Cross-study synthesis then depends on explicit lift over, harmonized representation rules, and re-annotation steps that keep variants comparable across versions [67]. Reference choice, therefore, belongs in the reproducibility record, not as background metadata. Beyond stating the reference and annotation versions, studies should name the conversion tools and chain files used, define normalization rules (e.g., left alignment), and specify conventions for SV and non-reference representation, filtering, and merging. When these choices are left implicit, technical drift can be mistaken for biological disagreement once cohorts and reference versions are combined [58,62].

Strategically, pangenomes and graphs move complex-trait analysis beyond SNP-only workflows toward joint modeling of SNPs and SVs. A unified representation reduces mapping and localization bias and stabilizes cross-lineage comparisons when the candidate mechanism is structural [48,61,68]. For breeding translation, representation advances also need matching inference infrastructure. High-quality references paired with large haplotype panels improve imputation from SNP chips or low-coverage data to sequence-level resolution, enabling fine mapping and genetic evaluation under cost constraints. As panels such as PHARP continue to expand, aligning cohorts under shared coordinates becomes easier, and the results become more reusable across studies, rather than being tied to a single platform or reference version [69,70].

Despite their advantages, graph-based and pangenome approaches require substantially greater computational resources and storage than linear-reference pipelines, and differences in graph construction and variant representation limit standardization across studies. Moreover, adoption in commercial genomic evaluation programs remains constrained by compatibility with SNP-chip platforms, imputation procedures, and routine quality-control practices. Taken together, these reference strategies present distinct advantages and practical trade-offs in pig genomics. To provide practical guidance for selecting reference strategies in pig genomics, a comparative summary of linear, breed-matched, and pangenome-based references is presented in Table 2.

## 3. Sequencing-Based Reconstructions of Evolutionary History in *Sus*

This section asks not only where domestic pigs originated, but how the lineage structure seen today was assembled across time and space. Human migration, trade, and animal movement repeatedly reshaped husbandry and kept managed herds in long contact with wild boar. Under chronic backcrossing and admixture, lineages were repeatedly reconfigured across periods, producing the regional ancestry mosaics seen in modern genomes.

Earlier reconstructions relied on mtDNA, sparse nuclear loci, and microsatellites. These markers captured broad divergence patterns, but they struggled once the dominant processes were repeated dispersal and sustained gene flow. In that setting, inference can be highly sensitive to marker choice and sampling, and different studies can tell different stories from the same underlying history. Whole-genome resequencing, time-stratified ancient genomics, and haplotype-resolved inference have shifted the field from plausible narratives to directly testable scenarios that can be cross-validated across datasets.

**Figure 2:**
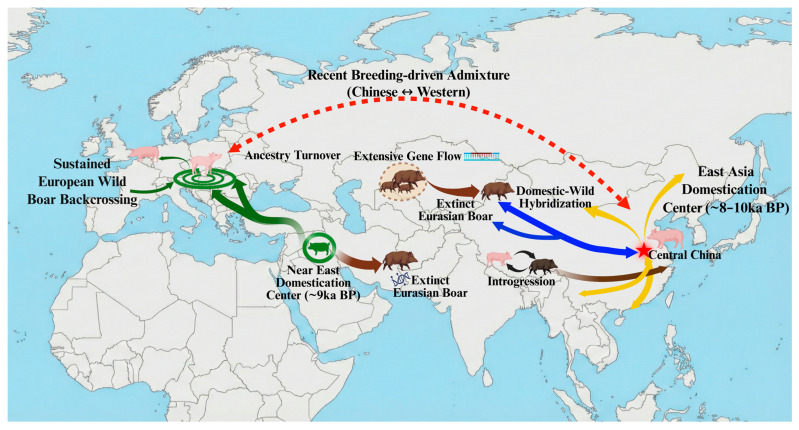
Global evolutionary history of *Sus*: a network perspective on domestication and gene flow. Note: The schematic summarizes major geographic regions and temporally layered processes shaping pig domestication and dispersal. Arrows represent gene flow and population interactions: green arrows indicate the Near East-to-Europe dispersal of early domestic pigs accompanied by sustained introgression from European wild boar into domestic populations; yellow arrows denote regional expansion of East Asian domestic pigs; blue arrows represent domestic–wild hybridization and bidirectional introgression between domestic pigs and wild boar; brown arrows indicate extensive gene flow among Eurasian wild boar lineages, including contributions from extinct or unsampled ancestral populations; and red dashed arrows denote recent breeding-era admixture between Chinese and Western domestic pigs. Additional annotations highlight ancestry turnover in Europe and episodes of hybridization and introgression across Eurasia. The red star marks Central China as a key domestication region.

Figure 2 summarizes the major regions and time windows used throughout this section. It summarizes major geographic theaters and key time windows and maps dispersal, admixture, and ancestry turnover events associated with changes in human management. This network interpretation is supported by multiple lines of analysis, including f-statistics-based tests of admixture, admixture graph modeling, haplotype-sharing analyses, and demographic inference methods that together reveal gene flow, ancestry mosaics, and temporally structured population divergence.

### 3.1. From Sparse Markers to Time-Stratified Ancient Genomes

Before high-throughput sequencing, *Sus* history was inferred mainly from mtDNA, limited nuclear fragments, and microsatellites. These data were sufficient for broad structure, but they often could not discriminate alternative domestication scenarios under recurrent movement and persistent gene flow, and could overemphasize maternal or local signals [71,72]. Whole-genome panels of modern pigs and wild boar reduced this ambiguity by placing inference in a consistent genome-wide variant background.

Ancient DNA added the decisive temporal dimension. Time-stratified archaeogenomics anchors observations to dated time points, making lineage turnover and gene exchange less dependent on indirect inference from present-day samples alone [73]. Because ancient DNA is fragmented and damage-prone, robust conclusions require authenticity checks, explicit damage modeling, and strict contamination control [74,75,76].

Europe illustrates how the temporal axis changes interpretation. Ancient genomes support the introduction of Near Eastern domestic pigs with early farming, followed by near genome-wide ancestry turnover driven largely by local wild boar contributions, so modern European domestic pigs retain only limited Near Eastern ancestry [14]. Population genomic analyses further show that gene flow and backcrossing vary spatially with ecological and management contexts [77]. This makes it possible to test, rather than assume, whether a region is better explained by repeated introductions or by sustained local backcrossing. Later breeding era movements also left genome-wide mosaics consistent with documented introductions and admixture [78,79]. Archaeogenomic results from Northwestern Europe underscore that husbandry strategies differed across sites, which is consistent with multiple coexisting pathways rather than a single standardized model [80].

In East Asia, time-stratified ancient genomes provide a similarly calibratable scaffold for dispersal and interaction, aligning shifts in genetic structure with human migration, trade, and husbandry change [73,81]. Whole-genome evidence for long-term divergence and regional adaptation in Eurasian wild boar further supplies an ecological reference for identifying source lineages, admixture partners, and conditions that favor replacement versus continuity [12,82]. Combining modern and ancient genomes makes it possible to estimate when transitions occurred and to distinguish replacement from continuity under explicit time constraints. That logic is what Figure 2 is meant to keep visible while the section moves across regions and time windows.

### 3.2. Multi-Stage Domestication and Regional Lineages: From Origins to Network History

Whole-genome and time-stratified ancient data increasingly support domestication as a network process rather than a simple branching tree. Domestication appears to involve multiple centers and recurrent incorporation of wild lineages, with persistent gene exchange and repeated reshuffling of ancestry [83,84,85]. Two early Neolithic centers are often discussed, the Near East (frequently linked to Anatolia) and East Asia (often placed in Central China), but early centers do not by themselves predict modern ancestry composition [83,84].

In Europe, Near Eastern domestic pigs were introduced early, yet sustained backcrossing with local wild boar drove near genome-wide turnover [14,80]. What varies is not the direction of gene flow, but its intensity and timing. Regional variation in management intensity likely explains heterogeneity in ancestry proportions and turnover pace [86]. In East Asia, ancient DNA supports regional continuity in maternal lineages alongside diversification [87], but whole-genome evidence is required to capture the admixture and replacement dynamics beyond uniparental markers [88,89].

Within China, large resequencing panels reveal strong differentiation among indigenous breeds and dense gene flow networks shaped by long-term husbandry, cross-regional movement, and historical hybridization [79,90]. Local breeds are, therefore, better interpreted as products of recombination and selection acting on mixed ancestry components under distinct environments and production systems, rather than as simple outcomes of linear divergence [20]. Recurrent gene exchange provides a second axis of evidence. Admixture with wild boar and more complex introgression can supply variation later acted upon by selection [84,91], and European datasets repeatedly detect domestic–wild admixture, with proposed adaptive contributions in some regions [86,92]. Modern improvement programs extend this network history into recent times, leaving bidirectional admixture between Chinese and Western pig populations [79,93,94]. Island systems, including Wallacea and Oceania, further highlight repeated introductions and renewed contact as recurring features of dispersal history [95].

Sex chromosomes offer an additional perspective. Pig Y haplotypes show patterns consistent with human-driven hybridization and selection, and expansions of specific paternal haplotypes align with recent breeding and breed introductions [78]. Comparing autosomes and sex chromosomes helps separate directional management from background demography and reduces the risk of over interpreting region specific patterns as purely ecological [79].

Overall, modern resequencing and time-stratified archaeogenomics favor a network-centered framework that integrates multi-stage domestication, regional lineage formation, and ancestry turnover [96]. Making gene flow and historical structure explicit is also the practical precondition for interpreting selection scans and trait associations across regions. It is easier to separate shared signals from lineage-specific ones when the time windows and routes are stated up front, as in Figure 2.

## 4. Genetic Diversity in Pigs

Pig genetic diversity reflects both deep demographic history and recent human-driven change. Long-term divergence among Eurasian wild boar lineages, shaped by climatic oscillations and geographic barriers, sets the baseline. Domestication and dispersal then added recurrent movement, backcrossing, and repeated reshuffling of ancestry, producing the region-specific mosaics observed in modern genomes. In the last decades, industrial breeding and global germplasm exchange have accelerated short-term change. Commercial lines tend to converge under strong selection, while local breeds increasingly serve as reservoirs of variation, including alleles relevant to adaptation and resilience.

This section moves from population structure to reusable resources and then to metrics that connect diversity to management. We first outline the global structure with a focus on Chinese indigenous pigs. We then summarize how harmonized coordinates and high-quality variant catalogues support cross-cohort comparison, imputation, SV mapping, and functional interpretation. Finally, we introduce inbreeding, runs of homozygosity (ROH), and mutational load as quantitative indicators of genetic health, emphasizing the need to interpret them against explicit demographic baselines.

### 4.1. Global Structure and Chinese Indigenous Resources

Local breeds and regional ecotypes remain widespread and often retain variation relevant to adaptation, fertility, resilience, and product quality [90]. Commercial production, by contrast, depends on a small number of globally distributed breeds and their crossbred composites. Three-way systems such as Duroc–Landrace–Yorkshire improve productivity, but sustained selection and large-scale germplasm movement also reshape structure and effective population size. A common contemporary pattern is, therefore, the coexistence of local resources with convergence among commercial populations [13,97]. China illustrates the scale of indigenous resources, with 147 breeds registered in 2024, about 23.5% of an estimated global total of 625 [98].

Global diversity does not collapse into a simple East–West contrast. Glacial cycles produced deep divergence among Eurasian wild boar lineages [82], yet domestication and dispersal repeatedly rewired haplotypes through migration and backcrossing, enabling rapid ancestry reshuffling and, in some cases, ancestry turnover in domestic populations [26]. It is, therefore, useful to separate two time layers in the interpretation: deep demographic history as a baseline and recent breeding plus industrial globalization as a fast perturbation capable of reordering structure over decades [89].

Chinese indigenous pigs contribute not only through breed richness but also through ecological breadth and historical depth. They span environments from tropical regions to cold high latitudes, and reflect heterogeneous management and selection histories, with differentiation in traits such as body size, fat deposition, fertility, roughage tolerance, and environmental adaptation. At the same time, commercial introductions in recent decades produced uneven European ancestry introgression across regions, complicating how local lineages connect to modern production systems [99]. Introgression is not uniformly detrimental. It can carry haplotypes with favorable effects, including signals linked to fertility and immune traits [100], and recent work also summarizes bidirectional introgression between Chinese and European populations on a broader scale [101].

Large whole-genome resources are turning these patterns into reusable infrastructure. The 1000 Chinese Indigenous Pig Genomes Project (50 local populations; 1011 individuals) provides a shared coordinate space for evaluating lineage relationships and demographic history, and supports data-driven conservation units and utilization strategies [102,103,104]. Diversity resources also matter beyond food production. Miniature pigs and laboratory strains are widely used in biomedicine and drug safety evaluation, and improved references and population resources strengthen standardization and traceability when these lineages serve as disease models and functional validation platforms [40,105].

### 4.2. Variant Catalogues and a Functional Annotation Framework

Mechanistic interpretation requires variant catalogues that remain comparable across breeds, together with an annotation framework that links variants to function and regulation. Haplotype reference panels and genotype imputation have become essential because they align heterogeneous SNP-chip platforms and cohorts to sequence-level representation, reducing platform-driven bias and improving cross-study comparability [69,106]. SWIM improved chip-to-sequence imputation, while PHARP 4.0 expanded the sample size and breed coverage, enabling broader cross-lineage comparison and fine mapping under shared coordinates [70,106].

As harmonization becomes routine, limitations of SNP-centric workflows are increasingly visible. Structural variation is being incorporated through large SV maps that improve cross-breed comparisons and expand candidate-variant prioritization [107,108,109]. Variant interpretation is also moving beyond a simple coding versus non-coding split toward criteria that combine evolutionary constraint, regulatory evidence, and tissue or cell-type specificity [110,111].

Regulatory resources help convert diversity patterns into testable mechanisms. PigGTEx links population variation to transcriptional regulation through multi-tissue eQTL and sQTL maps, supporting regulatory chains that connect elements, target genes, and tissue-specific effects [112]. Integration of PigGTEx with large-scale GWAS and meta-analyses is beginning to yield reusable workflows, including in reproductive traits [113,114,115]. PigBiobank further reduces barriers for reuse by organizing multi-cohort GWAS and functional resources in a searchable platform, facilitating joint analyses that connect diversity, traits, and mechanisms [116].

Finally, mutational load provides a quantitative view of the longer-term genetic costs of breeding. It connects effective population size, ROH structure, and the frequency spectrum of putatively deleterious variants [117,118]. Closed populations under strong selection often show a longer ROH and higher genomic inbreeding, while local breeds more often retain fragmented homozygosity and higher polymorphism. This shifts the probability that rare or recessive deleterious alleles become homozygous in high ROH backgrounds, with potential fitness costs [117,119]. Because purging and demographic history can reshape deleterious-allele frequencies, genetic cost should be interpreted on an explicit time scale and demographic baseline rather than through any single summary statistic [118,120].

## 5. Genetic Dissection of Major Economic Traits in Pigs

With genome-wide datasets, trait analysis increasingly prioritizes replicable association signals and their regulatory context rather than isolated candidate-gene claims. The central challenge is now portability: which signals replicate across breeding objectives and genetic backgrounds, and which are population-specific because of history, selection, and management. Functional annotation and tissue-resolved regulatory maps increasingly allow noncoding signals to be interpreted through regulatory elements and target genes, rather than relying on nearest-gene heuristics.

### 5.1. Growth and Carcass Composition

Growth rate, feed efficiency, and carcass composition remain core targets in commercial breeding because they determine output per unit time [121,122]. Their architecture typically combines a small set of larger-effect loci with a broad polygenic background, and leading signals can vary with breed history and selection direction [123]. Several classic loci remain informative because they connect association to plausible biology. A regulatory mutation in *IGF2* that affects postnatal expression is associated with increased muscle growth and lean percentage, consistent with altered tissue-specific expression patterns [93]. A missense variant in *MC4R* is repeatedly associated with coordinated shifts in growth and fat deposition, consistent with coupling between intake regulation and body composition [124].

Other loci recur across datasets but are often more background-dependent. Variants near *HMGA1* have been associated with body weight and body length, and are frequently discussed as candidates shaping growth trajectories [125,126]. *LEPR* commonly appears for backfat thickness and feeding-related traits, consistent with a recurring role in intake regulation and fat deposition across populations [127,128]. Functional variation in *NR6A1* is associated with an increased vertebral number and downstream changes in body and carcass length, illustrating how discrete genetic changes can translate into yield-relevant morphology [129].

Large GWAS and meta-analyses generally map daily gain, backfat thickness, lean percentage, and loin muscle area to multiple clusters of small-to-moderate effect. The same trait can show locus turnover across populations, and effect directions can differ [123,130]. This makes cross-population synthesis essential for separating shared from population-specific signals [130]. Carcass composition is also intrinsically composite, integrating fat deposition, muscle accretion, skeletal development, and visceral allocation, so loci can influence correlated endpoints in different ways [130,131]. In practice, backfat thickness often yields more stable signals, while loin muscle area and lean percentage are more sensitive to genetic background, slaughter protocols, and measurement heterogeneity. This keeps standardized phenotyping and cross-cohort validation central [121,128,131].

A recent example illustrates what a more complete evidence trail can look like. In a Bama Xiang pig × Landrace F1 hybrid population, a study combining GWAS and selection scans identified a chromosome 12 variant associated with backfat thickness (chr12:52,370,063 G > A) and highlighted *ALOX15* as a likely QTL gene. Haplotype–phenotype analyses showed a clear differentiation of favorable and unfavorable haplotypes between high- and low-backfat groups [132]. The value here lies in specificity: a defined variant, consistent haplotype effects, and selection evidence pointing to the same region.

For application, the multi-endpoint nature of growth and carcass traits favors joint models and multi-trait genomic prediction to improve accuracy while limiting adverse correlated responses [122]. For feed efficiency, systematic reviews and meta-analyses can distill regions that recur across cohorts and measurement systems [133]. Divergent selection experiments for residual feed intake also clarify why joint modeling of intake, efficiency, carcass traits, and meat quality is needed to make trade-offs explicit [134]. Mechanistic prioritization increasingly draws on tissue expression, cis-eQTL, and chromatin accessibility to narrow candidates and highlight the regulatory axes linked to muscle accretion, adipogenesis, and insulin signaling [135,136]. Commercial analyses are also beginning to analyze production together with immune and robustness traits to identify shared nodes that matter for both performance and health [137]. In practice, genomic selection captures much of the polygenic background, while a small number of replicable loci can be managed more directly when the effects are stable and trade-offs are understood [93,138].

### 5.2. Meat Quality and Muscle Metabolism

Meat quality includes intramuscular fat, tenderness, drip loss, color, ultimate pH, and flavor-related metabolites. Compared with growth traits, these phenotypes are more sensitive to slaughter procedures, transport stress, and batch effects, and their signals are often more context-dependent across genetic backgrounds, production systems, and slaughter stages [139,140]. Interpretation increasingly treats meat quality as an integrated physiological outcome shaped by energy metabolism, redox balance, membrane properties, and ion homeostasis [139,141].

Several loci have clear mechanistic links. *PRKAG3* (*RN*) influences muscle glycogen metabolism and postmortem acidification, shifting pH, drip loss, and processing quality [142]. The HAL locus in *RYR1* is linked to stress susceptibility and pale, soft, exudative meat risk, implicating calcium homeostasis and excitation-contraction physiology [143]. Unfavorable alleles increase the risk of paler color and higher drip loss, and RYR1 remains a classic marker used in quality control and selection [144].

Beyond these major loci, recurring signals often map to lipid deposition and fatty acid composition. *SCD*, genes such as *FASN* and *ACACA*, and members of the FABP family are repeatedly implicated, consistent with selection on lipid synthesis, transport, and storage networks within muscle [145,146]. In commercial populations, many associations appear polygenic and regulatory in nature and often converge on mitochondrial function, oxidative stress responses, and energy metabolism at the pathway level [139,147].

This helps explain the growing use of designs that integrate GWAS with transcriptomics or metabolomics in matched tissues and stages, especially for process traits such as drip loss, where membrane integrity, ion transport, and redox state recur as candidate axes [148,149]. With better standardization and integrated multi-omics, it becomes easier to distinguish variation driven mainly by slaughter processes from variation reflecting the baseline muscle metabolic state [150]. Breed-comparison studies that connect genes to metabolites also help shift the interpretation from isolated hits to pathways and actionable nodes [151].

As multi-reference assemblies and SV scanning become more common, some hypotheses now point to specific regulatory structures. A deletion SV in the first intron of *AR*, near an active promoter region, has been proposed as a regulatory change affecting *AR* expression and contributing to color-related phenotypes [152]. Because the causal chain linking SV, gene expression, and phenotype can depend strongly on tissue matching and developmental stage, this signal is best treated as a prioritized candidate until targeted functional tests establish causality.

Breeding decisions increasingly consider meat quality together with robustness traits, as welfare requirements and reduced antimicrobial use change the constraints under which quality must be maintained [137]. Phenotypic standardization and cross-batch comparability remain limiting steps, keeping large cohorts with unified protocols and multi-cohort validation essential [140]. As regulatory atlases mature, noncoding signals mapping to muscle-specific elements should become easier to validate and deploy in selection.

### 5.3. Reproduction and Litter Outcomes

Reproductive traits include the total number born, number born alive, survival to weaning, age at first farrowing, and maternal behavior. They typically show low heritability, strong environmental noise, and a complex correlation structure [153,154]. Large-scale recording and long-term data are, therefore, essential, and models must account for parity, farm, and management effects to reduce non-genetic variance [153]. Genetic parameters can also vary across parities, so parity-specific modeling can be appropriate [155].

Signals distribute across ovarian function, implantation, uterine capacity, placental development, and immune tolerance, and single variants rarely explain large shares of variance [154]. For composite outcomes such as litter size or weaning weight, component phenotypes can improve interpretability. Teat number, a proxy for nursing capacity, has produced multiple reproducible signals in GWAS and meta-analyses [48]. Male fertility is also increasingly analyzed under artificial insemination. A multi-breed meta-GWAS integrating PigGTEx identified reproducible loci and regulatory candidates for semen volume, sperm number, and motility, linking reproductive phenotypes to regulatory QTL and candidate genes [156].

Some loci remain operationally useful when they replicate across settings. *ESR1* is a classic example, with favorable alleles associated with increased litter size or number born alive across multiple studies [157,158]. Studies that combine genomic selection and GWAS for litter traits in commercial populations are also increasing, which places the evidence closer to the real selection environment [159].

Regulatory anchoring is improving. Using cross-population meta-GWAS with PigGTEx annotation, one study proposed UGT2B31 as a highly expressed liver gene with a cis-eQTL (rs344053754) that may regulate expression and influence litter weight at weaning and related outcomes. In the same framework, gestation-length signals were more enriched in the ovary, supporting tissue-specific interpretation [114]. Joint modeling of intermediate phenotypes, including body weight dynamics, backfat, intake and energy traits, or blood and metabolomic markers, can also reveal variants acting through the maternal metabolic state and improve early prediction and portability [160,161].

In practice, genomic selection remains the most reliable path for steady gain under polygenicity, but it performs best when inbreeding and correlated responses are managed explicitly [162,163,164]. Reproduction is genetically correlated with growth, body composition, and health, so single-trait emphasis can create undesirable outcomes, including larger litters with lower survival or compromised sow condition [165]. Recent work treats piglet survival and maternal robustness as primary targets alongside litter size and uses finer phenotyping to partition losses by biological stage, improving both evaluation and mechanistic alignment with time- and tissue-resolved regulatory maps [112,116,136,165].

### 5.4. Disease Resistance

As disease pressure rises and antimicrobial use is reduced, disease resistance and robustness are increasingly treated as primary breeding objectives. Compared with growth traits, resistance phenotypes depend more on challenge experiments or detailed field records and are strongly affected by pathogen heterogeneity, immune status, and management. This keeps phenotyping design and model specification central, including an explicit representation of exposure and batch effects [166]. Where possible, resistance, meaning pathogen control, and tolerance, meaning maintained performance under infection, should be separated because they can have different genetic bases and different epidemiological consequences.

PRRS illustrates both the presence of major loci and the limits of single-locus thinking. Host response shows substantial genetic variation, and GWAS mapped a key SSC4 QTL represented by the WUR marker. A large polygenic background remains, so breeding populations often follow a major-locus plus polygenic architecture [167]. Validation suggests that favorable WUR alleles can be used without large penalties to production, supporting routine selection [168,169]. Mechanistic work connected this region to immune regulation involving candidates such as *GBP5* [170].

*CD163* editing is a clear example of functional translation. Removal of the SRCR5 domain preserves macrophage function while resisting PRRSV in cellular models, and pigs lacking CD163 SRCR5 show resistance to PRRSV-1 in challenge experiments [171,172]. Population-level evaluations suggest no obvious adverse consequences for growth and meat quality, making this one of the most advanced experimental cases reported so far [173].

For African swine fever (ASF), comparative hypotheses implicate immune signaling. Variants in *RELA* (NF-kB p65) have been proposed as contributors to tolerance in warthogs, providing a candidate axis for domestic pigs [174]. However, introducing warthog *RELA* variants into domestic pigs did not reproduce tolerance on its own, which argues against single-axis sufficiency [175]. Evidence from African local pig populations links *RELA* polymorphism and upregulation to outcome, but transferability still depends on controlled infection models and functional tests that establish directionality [176]. More broadly, ASF has become a major disease threat in global pig production, prompting increasing interest in host genetic determinants of disease outcome [177]. Emerging evidence suggests that ASF tolerance is shaped by complex interactions among immune regulation, host genetic background, and environmental context, indicating a polygenic architecture rather than single-locus control [174]. These observations highlight the need for integrative genomic and experimental approaches to identify breeding-relevant markers and improve the translation of ASF-related genetic findings into practical selection strategies.

In practice, disease improvement often combines broad genomic selection for robustness with the targeted use of a small number of high-confidence causal nodes when functional evidence is strong and side effects are manageable, as in *CD163* for PRRSV [166,171,172,173]. A comparative summary of the representative loci, candidate genes, and pathways is provided in Table 3.

## 6. Summary and Outlook

Pig genomics is now being pulled into one chain of evidence: genome representation, population history, and trait mechanism. Better linear references, breed-matched assemblies, and graph-based pangenomes have made analyses in repeat-rich and SV-prone regions less biased, which is why cross-breed comparisons, ancestry inference, and fine-mapping have become more stable when cohorts share a coordinate space. Notably, genomic resources have progressed from single linear references to multi-assembly and graph-based representations, improving structural variant detection and cross-study comparability (Section 2). Time-stratified ancient genomes add a dated axis that keeps reinforcing a network history of *Sus*, with repeated domestication and dispersal, persistent gene flow, and, in some regions, substantial ancestry turnover. Trait genetics is also shifting in what counts as a useful result, not just where a signal sits, but which regulatory change acts in which tissue or stage, and whether that effect holds across breeding contexts. Genetic dissection of economically important traits reveals both major-effect loci and polygenic architectures relevant to production performance, reproduction, and disease resilience (Section 5).

What holds the field back is not another assembly, but consistency when moving across representations. Results can drift because coordinates, SV definitions, and normalization rules are handled differently, so disagreements appear that look biological but are often methodological. The most practical way forward is to make translation steps explicit and standardized: coordinates, SV normalization, and liftover or conversion should be reported as core methods; pangenomes and graphs should connect cleanly to the tools breeding programs already use at scale, especially imputation, high-throughput genotyping, and routine QC; and validation should begin with a specific element-to-gene hypothesis tested in matched tissues and stages, with readouts that let others follow the chain from perturbation to molecular change and, where possible, to organism-level outcomes.

In short, the next step is portability. If results remain comparable across studies and representations and validation stays traceable and context-matched, pig genomics can keep delivering genetic gain while treating diversity retention as a routine constraint rather than an after-the-fact repair. Building on this perspective, improved portability has direct implications for long-term genomic selection programs, as stable variant definitions and effect estimates across assemblies and cohorts allow breeding values to remain comparable over time, reduce reparameterization when reference systems change, and facilitate the joint use of historical and newly generated datasets. At the same time, several open questions remain, including the contribution of structural variants and regulatory elements to long-term selection response, practical integration of graph-based variation into genomic prediction, and the challenge of balancing sustained genetic gain with diversity retention under changing production environments. From a practical standpoint, improving reproducibility in pig genomics requires the explicit reporting of reference assemblies and annotation versions, standardized variant representation and coordinate translation procedures, and validation strategies based on matched tissues and developmental contexts. Ensuring compatibility between emerging pangenome resources and routine breeding pipelines, together with replication across independent cohorts, will be critical for maintaining cross-study comparability and supporting reliable long-term genetic improvement.

## 7. Conclusions

Pig genomics has developed rapidly in recent years, moving from the construction of reference genomes toward a broader understanding of evolutionary history and the genetic basis of economically important traits. Improvements in genome assemblies, together with the growing availability of breed-specific genomes and pangenome resources, have expanded the ability to capture structural and non-reference variation and improved the resolution of genomic analyses. At the same time, whole-genome sequencing and ancient DNA studies have reshaped our understanding of the complex demographic history of *Sus*, highlighting repeated domestication processes, gene flow, and regional ancestry turnover. Research on production traits is also shifting from the simple localization of association signals toward identifying the regulatory and biological mechanisms that link genetic variation to phenotype. Ensuring comparability across reference systems and analytical pipelines will remain essential for reliable interpretation and cross-study integration. Continued integration of genomic resources with functional annotation and gene expression data will further improve the interpretation of complex traits and support the effective use of genomic information in pig breeding and conservation.

## Figures and Tables

**Table 2:** Practical consequences of different genome reference strategies in pig genomics.

Reference Strategy	Key Advantages	Main Limitations	Implications for Genomic Evaluation	Recommended Breeding Application
Linear reference	Stable coordinate system; tool compatibility	Reference bias, limited SV resolution	High reproducibility; efficient large-scale genomic prediction	Routine genomic selection; SNP-chip–based evaluations
Breed-matched assembly	Reduced reference bias; improved variant discovery	Limited crossbreed portability	Enhanced within-breed prediction accuracy; fine-mapping resolution	Breed-specific genomic analyses; targeted selection programs
Pangenome/graph	Comprehensive variant representation; improved SV detection	High computational demand; limited standardization	Improved structural variant modeling but increased analytical complexity	Research-driven trait discovery; exploratory breeding analyses

Abbreviation: SV, Structural variant; SNP, Single-nucleotide polymorphism.

**Table 3:** Candidate genes and pathways underlying major economic traits in pigs.

Trait Category	Representative Traits	Key Genes/Loci	Biological Mechanisms/Pathways	Key References
Growth and carcass composition	ADG, feed efficiency, backfat thickness, loin eye area, lean %, carcass length	*MC4R/LEPR*; *IGF2*; *NR6A1*; +polygenic loci	Energy balance/appetite; adipogenesis; IGF signaling; skeletal patterning	[127,129,133,178]
Meat quality and muscle metabolism	Intramuscular fat, pH/acidification, drip loss, color, tenderness	*PRKAG3*; *RYR1*; loci for pH/WHC/colour	Post-mortem glycolysis; water holding; proteolysis; muscle energy metabolism	[139,149,151,179]
Reproduction and maternal performance	Litter size, piglet survival, weaning litter weight, teat number, maternal performance	Polygenic signals; recurrent regions; rs344053754-UGT2B31	Composite/low-heritability; ovulation–uterus capacity–survival; variant–gene–tissue links	[48,114,154,165]
Disease resistance and robustness	PRRS resilience, general robustness; ASF tolerance-related outcomes	SSC4 WUR/GBP5; *CD163*; RelA/NF-κB	Standardized challenge/field phenotyping; GWAS + functional validation; immune regulation/tolerance	[173,176,180,181]

## Data Availability

No new data were created or analyzed in this study. Data sharing is not applicable to this article.

## Supplementary Materials

The following supporting information can be downloaded at https://www.mdpi.com/article/10.3390/biology15050447/s1: Table S1: Genome size of representative pig reference assemblies.
